# Supplementary material for: Retinopathy among women with hypertensive disorders of pregnancy attending hospitals in Mbarara city, south-western Uganda: a cross-sectional study
Source: BMJ Open. 2023 Oct 10;13(10):e076365. doi: 10.1136/bmjopen-2023-076365 (PMC10565131; doi:10.1136/bmjopen-2023-076365)
Supplement: Author's manuscript [file bmjopen-2023-076365.draft_revisions.pdf]

BMJ Open is committed to open peer review. As part of this commitment we make the peer review history of every article we publish publicly available.

When an article is published we post the peer reviewers' comments and the authors' responses online. We also post the versions of the paper that were used during peer review. These are the versions that the peer review comments apply to.

The versions of the paper that follow are the versions that were submitted during the peer review process. They are not the versions of record or the final published versions. They should not be cited or distributed as the published version of this manuscript.

BMJ Open is an open access journal and the full, final, typeset and author-corrected version of record of the manuscript is available on our site with no access controls, subscription charges or pay-per-view fees (http://bmjopen.bmj.com).

If you have any questions on BMJ Open's open peer review process please email info.bmjopen@bmj.com

BMJ Open

Retinopathy among women with hypertensive disorders of pregnancy attending hospitals in Mbarara city, south-western Uganda

Journal:	*BMJ Open*
Manuscript ID	bmjopen-2023-076365
Article Type:	Original research
Date Submitted by the Author:	05-Jun-2023
Complete List of Authors:	Zamaladi, Ibrahimu; Mbarara University of Science and Technology, Department of Ophthalmology<br>Ruvuma, Sam; Mbarara University of Science and Technology, Department of Ophthalmology<br>Mceniery, Carmel M. ; University of Cambridge<br>Kwaga, Teddy; Mbarara University of Science and Technology, Department of Ophthalmology<br>Wilkinson, Ian; Division of Experimental Medicine and Immunotherapeutics, Department of Medicine, University of Cambridge; Cambridge Clinical Trials Unit,<br>Atwine, Daniel; Soar Research Foundation, Department of Clinical Research; Mbarara University of Science and Technology, Department of Community Health<br>Lugobe, Henry Mark; University of Cambridge; Mbarara University of Science and Technology, Department of Obstetrics and Gynecology
Keywords:	Hypertension < CARDIOLOGY, Maternal medicine < OBSTETRICS, Medical ophthalmology < OPHTHALMOLOGY

SCHOLARONE™
Manuscripts

I, the Submitting Author has the right to grant and does grant on behalf of all authors of the Work (as defined in the below author licence), an exclusive licence and/or a non-exclusive licence for contributions from authors who are: i) UK Crown employees; ii) where BMJ has agreed a CC-BY licence shall apply, and/or iii) in accordance with the terms applicable for US Federal Government officers or employees acting as part of their official duties; on a worldwide, perpetual, irrevocable, royalty-free basis to BMJ Publishing Group Ltd ("BMJ") its licensees and where the relevant Journal is co-owned by BMJ to the co-owners of the Journal, to publish the Work in this journal and any other BMJ products and to exploit all rights, as set out in our licence.

The Submitting Author accepts and understands that any supply made under these terms is made by BMJ to the Submitting Author unless you are acting as an employee on behalf of your employer or a postgraduate student of an affiliated institution which is paying any applicable article publishing charge ("APC") for Open Access articles. Where the Submitting Author wishes to make the Work available on an Open Access basis (and intends to pay the relevant APC), the terms of reuse of such Open Access shall be governed by a Creative Commons licence – details of these licences and which Creative Commons licence will apply to this Work are set out in our licence referred to above.

Other than as permitted in any relevant BMJ Author's Self Archiving Policies, I confirm this Work has not been accepted for publication elsewhere, is not being considered for publication elsewhere and does not duplicate material already published. I confirm all authors consent to publication of this Work and authorise the granting of this licence.

For peer review only

Retinopathy among women with hypertensive disorders of pregnancy attending hospitals in Mbarara city, south-western Uganda

Ibrahimu Zamaladi[1], Sam Ruvuma [1], Carmel M. Mceniery[2] Teddy Kwaga[1], Ian B. Wilkinson[2], Daniel Atwine[3,4], Henry Mark Lugobe[2,5]*

[1]Department of Ophthalmology, Mbarara University of Science and Technology, Mbarara, Uganda.

[2]Division of Experimental Medicine and Immunotherapeutics, University of Cambridge, Cambridge, UK

[3]Department of Clinical Research, Soar Research Foundation, Mbarara, Uganda

[4]Department of Community Health, Mbarara University of Science and Technology, Mbarara, Uganda.

[5]Department of Obstetrics and Gynecology, Mbarara University of Science and Technology, Mbarara, Uganda.

*Corresponding author:

Henry Mark Lugobe

PO Box 1410, Mbarara

henrylugobe@must.ac.ug

For peer review only

Word count: 2389

Keywords: Retinopathy, Hypertensive disorders of pregnancy, Pregnancy, Uganda

For peer review only - http://bmjopen.bmj.com/site/about/guidelines.xhtml

Abstract

Objective: Retinopathy is one of the complications occurring among women with hypertensive disorders of pregnancy. We sought to determine the prevalence and factors associated with retinopathy among women with hypertensive disorders of pregnancy in southwestern Uganda.

Design: This was a hospital based cross-sectional study from November 2019 to March 2020.

Setting: Three selected hospitals in Mbarara city, south-western Uganda,

Participants: The study included all pregnant women with hypertensive disorders of pregnancy.

Primary and secondary outcome measures: The participants were screened for retinopathy using a fundus camera. Data on participant's socio-demographics, obstetrics, and medical factors were collected. The prevalence of retinopathy was determined and multivariable logistic regression was used to determine the independent factors associated with retinopathy.

Results: A total of 216 women with hypertensive disorders of pregnancy were enrolled in this study. The prevalence of retinopathy was 60.1% (130/216). The commonest retinal lesions were Grade 1 retinopathy (narrowing of arterioles) accounting for 86.9% (113/130), Grade 3 (retinal hemorrhages) was present in 10% (13/130) of women, and Grade 4 (papilledema) in 3% (4/130). In an adjusted analysis, severe hypertension was significantly associated with retinopathy (aOR=2.8; 95%CI: 1.36, 5.68). Grandmultigravida women were also associated with retinopathy (aOR=2.4; 95%CI: 0.99, 5.72) with a tendency towards significancy, p=0.051.

Conclusions: In our study, retinopathy was common among women with hypertensive disorders of pregnancy. Women presenting with severe hypertension were likely to have retinopathy. There is a need to integrate screening for retinopathy in the care cascade of women with hypertensive disorders of pregnancy.

For peer review only - http://bmjopen.bmj.com/site/about/guidelines.xhtml

Strengths and limitations of this study

- A portable non-mydriatic fundus camera was used to examine for retinopathy.
- Blood pressure was only done at one time point.
- Routine screen the participants for diabetes or glucose intolerance during their pregnancy was not done.
- Biochemical tests that determine hypertension with end organ damage were not done as we relied only on the blood pressure.

For peer review only

For peer review only - http://bmjopen.bmj.com/site/about/guidelines.xhtml

Introduction

Retinopathy in pregnancy is a term that defines the retinal pathologies seen uniquely in pregnancy or more commonly in conditions that may worsen or alter during pregnancy as a result of hematologic and metabolic change [1]. During pregnancy, elevated blood pressure poses various risks including placental abruption, intrauterine growth retardation, premature delivery, future cardiovascular diseases, and injury to other organs including the eye [2]. Ocular involvement is common in a majority of cases of hypertensive disorders of pregnancy. Common symptoms are blurry vision, photopsia, scotomas, and diplopia [3]. Visual disturbances and retinopathy in hypertensive disorders of pregnancy seem to be a frequent phenomenon [4]. The Global prevalence of hypertensive disorders of pregnancy ranges between 5-10% and fundus changes are seen in 40-100% of these patients [5]. In Africa, hospital-based prevalence of hypertensive disorders of pregnancy is 8% in Ethiopia [6] and 17% in Nigeria [7]. In Uganda, hypertensive disorders of pregnancy accounted for 12% of maternal deaths in 4 referral hospitals [8] while at Mbarara regional referral hospital they accounted for 22% of the maternal deaths [9].

Studies in Malaysia and India showed that 40% and 59% of women with hypertensive disorders of pregnancy respectively presented with retinal changes while in Nepal it was 14% [10]. In Uganda, a study done at Mulago National Hospital showed a prevalence of 16.5% of retinal changes in preeclampsia/eclampsia patients [11]. Retinopathy among women with hypertensive disorders of pregnancy is associated with fetal birth weight, serum uric acid, proteinuria, severe hypertension and maternal age[12-15].

However, there is a paucity of information on retinopathy among women with hypertensive disorders of pregnancy in sub-saharan Africa where hypertension in pregnancy is associated with significant maternal morbidity and mortality. We sought to determine the prevalence and factors associated with retinopathy among women with hypertensive disorders of pregnancy in south-western Uganda.

Materials and Methods

Study design and setting

We conducted a hospital-based, cross-sectional study of women with hypertensive disorders of pregnancy, at three selected hospitals in south-western Uganda from November 2019 to March

For peer review only - http://bmjopen.bmj.com/site/about/guidelines.xhtml

2020. These hospitals were Mbarara Regional Referral hospital, Mayanja Memorial hospital, and Divine Mercy hospital. Mbarara regional referral hospital is a government-owned referral hospital that offers free services as well as a teaching hospital for the medical school of Mbarara University of Science and Technology. The hospital conducts about 10,000 deliveries every year. Mayanja Memorial Hospital is a private hospital with a capacity of 60 beds while Divine Mercy Hospital is also a private hospital with a capacity of 22 beds. All these facilities offer antenatal, labor, delivery, and post-natal care services. These facilities receive referrals from sixteen districts in south-western Uganda.

We received ethical approval from the Mbarara University of Science and Technology Research Ethics Committee (NO 16/09-19). All the study participants provided written informed consent.

Study participants

Our study population included all pregnant women at ≥ 20 weeks of gestation with hypertensive disorders of pregnancy who were receiving care either as in-patients at the antenatal ward or as out-patients at the antenatal clinic. The blood pressure was measured at admission or during the antenatal clinic visit. Hypertension was defined as systolic blood pressure ≥140mmHg or diastolic blood pressure ≥ 90mmHg. Women with ocular diseases that prevented adequate fundus examination, such as cataracts, corneal opacities, and ocular trauma, were excluded from the study. All mothers presenting at the maternity ward or antenatal clinic had their blood pressure checked as part of routine clinical care and eligible participants were consecutively enrolled in the study.

Variables and data sources

Hypertensive disorders of pregnancy were classified into eclampsia, pre-eclampsia, , gestational hypertension, chronic hypertension, and chronic hypertension with superimposed preeclampsia, according to consensus definitions [16]. All pregnant women who had hypertensive disorders of pregnancy either diagnosed as inpatient or outpatient were screened for retinopathy. The primary outcome for our study was retinopathy and was determined using Keith and Wagner classification [17].

Grade 1: Mild generalized arterial attenuation, particularly of small branches, with broadening of the arteriolar light reflex.

For peer review only - http://bmjopen.bmj.com/site/about/guidelines.xhtml

Grade 2: Marked generalized narrowing and focal attenuation of arterioles associated with arterio-venous nipping/narrowing (Salus' sign).

Grade 3: Grade 2 changes plus copper-wiring of arterioles, banking of veins distal to arteriovenous crossings (Bonnet sign), tapering of veins on either side of the crossings (Gunn sign). Flame-shaped hemorrhages, cotton wool spots, and hard exudates are also present.

Grade 4: All changes of Grade 3 plus silver wiring of arterioles and papilledema.

Uncorrected visual acuity was measured using a Snellen chart and E-chart. Anterior segment examination was done using a torch while fundoscopy was done using both a direct ophthalmoscope (brand Keeler) after dilating the pupil with tropicamide 1%, and used a portable non-mydriatic fundus camera (Forus 3nethra non-mydriatic, Forus Health Pvt Ltd, Bengaluru, India). Fundus changes were recorded and all fundus photos were interpreted by a consultant retinal specialist of Mbarara University Referral Hospital Eye Center. The fundus pictures were graded for quality before KW grading.

The independent variables included socio-demographic characteristics (age, level of education, address, district of origin, and occupation), medical factors (known history of diabetes mellitus, hypertension), and obstetric factors (gravidity, severity of hypertension (systolic blood pressure ≥160mmHg and or diastolic blood pressure ≥100mmHg), and gestational age). At enrolment into the study, participants were interviewed and information was obtained using an interviewer-administered questionnaire.

Sample size calculation

We calculated a total sample size of 216 participants using the formula for cross-sectional sample size calculations of Kish Leslie with the following assumptions.

$n = (Z\alpha^2 p\,(1-p))/\delta^2$

Where

n= sample size estimate.

p= prevalence (16.5 %) as estimated by a study done at Mulago hospital [11]

δ= acceptable margin of error 5% (0.05).

$Z\alpha$= standard normal deviation at 95% Confidence Interval 1.96.

1-p= the probability of having retinopathy (1-0.17) = 0.83

$n = (1.96^2 \times 0.17(1-0.17)) / 0.05^2$

n= 216 participants

For peer review only - http://bmjopen.bmj.com/site/about/guidelines.xhtml

Statistical analysis

Data were entered into Microsoft excel 2010 and were imported into STATA 15.0 software (college station Texas USA) for analysis. Descriptive statistics were presented as means and standard deviations for continuous variables and proportions for categorical variables. The proportion of patients with retinopathy was calculated as the number of participants with retinal changes over the total number of study participants and expressed as a percentage with its corresponding 95% Confidence Interval (CI). Retinal changes were classified according to KEITH WAGNER classification into grade 1, grade 2, grade 3, and grade 4, and the proportion of each grade was presented in a frequency table. Bivariate analysis using pearson Chi-square test and logistic regression analysis was performed comparing participant factors with retinopathy. At bivariate analysis, variables with statistically significant association with retinopathy ($p<0.05$), those with $p<0.1$ and those with biological plausibility with retinopathy e.g age were included in the multivariable logistic regression model. A manual stepwise backward elimination was used to build the final model. The independent factors associated with retinopathy in the final multivariable model were reported together with their adjusted odds ratios and 95% confidence interval. The level of significance was $p<0.05$.

Results

Study Profile

A total of 5008 pregnant women attended Mbarara Regional Referral Hospital, Mayanja memorial Hospital, and Divine Mercy Hospital during the study period. Of these, 216 women with hypertensive disorders of pregnancy were consented and enrolled in the study. A total of 208 (96.3%), 6 (2.8%) and 2 (0.9%) were recruited from Mbarara Regional Referral Hospital, Mayanja Hospital and Divine Mercy Hospital respectively. Of the participants, 88.4% (191/216) had preeclampsia, 10.2% (22/216) had eclampsia and 1.4% (3/216) had gestational hypertension.

Participants' Characteristics

Table 1 shows the sociodemographic and medical characteristocs of women enrolled in the study. Participants had a mean age of 27.6±6.37 years with majority aged less than 35 years (81.7%), Ugandan (90.3%) and either unemployed or peasants (74.5%). Participants predorminantly had 37 or more gestational weeks (70.4%) and were either multigravida or

For peer review only - http://bmjopen.bmj.com/site/about/guidelines.xhtml

grand multigravida mothers (72%). One in four had severe hypertension (26.4%) at the time of the study. Majority of participants (214/216) had good uncorrected visual acuity of 6/6-6/18, and only 1/216 had severe visual impairment. A third of the participants presented with blurry vision. There was no case of complete blindness in our study.

Table 1: Baseline characteristics of participants

Characteristics		n (%)
Age in years, mean(SD)		27.6 (6.37)
Age category	<25	77 (35.4)
	25-34	100 (46.3)
	35-46	39 (18.1)
Nationality	Ugandan	195 (90.3)
	Non-Ugandan	21 (9.7)
Occupation	Unemployed	84 (38.9)
	Peasant	77 (35.6)
	Business	30 (13.9)
	Professional	17 (7.9)
	Laborer	8 (3.7)
Known history of diabetes mellitus	No	209 (96.8)
	Yes	7 (3.2)
Severe hypertension	No	159 (73.6)
	Yes	57 (26.4)
Gravidity	Primigravidas	61 (28.2)
	Multigravida	111 (51.4)
	Grand multigravida	44 (20.4)
Gestational age (weeks)	<37	64 (29.6)
	≥37	152 (70.4)
Ocular symptoms	No complaints	42(19.4)
	Blurry vision	71 (32.9)
	Reduced vision	7 (3.3)
	Photopsia	2 (0.9)
	Inability to focus	1 (0.5)
	Visual field defect	1 (0.5)
Visual acuity	6/6-6/18	214 (99.0)
	<6/18-6/60	1 (0.5)
	6/60-3/60	1 (0.5)

Prevalence of hypertensive retinopathy among pregnant women

Of the 216 women with hypertensive disorders of pregnancy attending hospitals in Mbarara city and enrolled in the study, 130 had retinopathy, giving a prevalence of 60.2% (95% CI: 53.5-66.5). No age-specific disparities where noted with regard to prevalence of retinopathy, p=0.136 as shown in Table 2.

For peer review only - http://bmjopen.bmj.com/site/about/guidelines.xhtml

Table 2. Prevalence of hypertensive retinopathy among pregnant women

Prevalence type	N	n	% (95% CI)	p value
Overall	216	130	60.2 (53.5-66.5)	NA
Age-specific				0.136
<25	77	44	57.1 (45.7-67.9)	
25-34	100	57	57.0 (47.0-66.5)	
35-46	39	29	74.4 (57.8-86.0)	

Of the 130 women with retinopathy, 113 (86.9%) presented with arteriolar narrowing. Severe retinopathy characterized by retinal hemorrhages and papilledema was also recorded. No one had Grade 2 retinopathy and no retinal detachment as shown in Table 3.

Table 3: Description of retinal lesions found among women with hypertensive disorders of pregnancy according to Keith and Wagner classification

Retinal lesions (n=130)		n (%)
Grade 1	Arteriolar narrowing	113 (86.92)
Grade 3	Dot and blot hemorrhages	6 (4.62)
	Flamme shape hemorrhages	3 (2.30)
	Cotton wool spots	2 (1.54)
	Exudates	2 (1.54)
Grade 4	Papilledema	2 (1.54)
	Maculoedema	2 (1.54)

Factors associated with retinopathy among women with hypertensive disorders of pregnancy

Table 4 shows results of bivariate and multivariate analysis. In bivariate analysis, the only factors that were significantly associated with retinopathy where gravidity (p=0.049) and severe hypertension (p=0.005).

In multivariate analysis analysis, severe hypertension was the only factor significantly associated with retinopathy after adjusting for age, gravidity and gestational age, p=0.005. The odds of having retinopathy where 2.8 times higher among women with severe hypertension as compared to those without, OR=2.8; 95%CI: 1.36, 5.68. Also, the odds of

For peer review only - http://bmjopen.bmj.com/site/about/guidelines.xhtml

having retinopathy were 2.4 times higher among grandmultigravida women as compared to those with multigravida women, OR=2.4; 95%CI: 0.99, 5.72, although this did not attain statistical significancy, p=0.051.

Table 4: Factors associated with retinopathy among women with hypertensive disorders of pregnancy.

Variable	Categories	Retinopathy n (%)	Crude OR (95% CI)	p-value	Adjusted OR (95% CI)	p-value
Age	<25	44 (57.1)	Ref	0.124	Ref	
	25-34	57 (57.0)	0.9 (0.55,1.81)		0.9 (0.42,1.72)	0.655
	35-46	29 (74.4)	2.2 (0.93,5.08)		1.5 (0.54,4.18)	0.435
Nationality	Ugandan	116 (59.5)	Ref	0.519		
	Non-ugandan	14 (66.7)	1.4 (0.53,3.53)			
Occupation	Unemployed	56 (66.7)	1.8 (0.62,5.11)	0.4723		
	Peasant	41 (53.3)	1.0 (0.35,2.90)			
	Business	19 (63.3)	1.5 (0.46,5.14)			
	Professional	9 (52.9)	Ref			
	Laborer	5 (62.5)	1.5 (0.27,8.27)			
Severe hypertension	No	87 (54.7)	Ref	0.005	Ref	
	Yes	43 (75.4)	2.5 (1.29, 5.01)		2.8 (1.36,5.68)	0.005
Gravidity	Multigravid	60 (54.1)	Ref	0.049	Ref	
	Primigravida	37 (60.7)	1.3 (0.69,2.47)		1.2 (0.61,2.54)	0.556
	Grandmultigravida	33 (75.0)	2.5 (1.17,5.55)		2.4 (0.99,5.72)	0.051
Gestational age	≥37	88 (57.9)	Ref	0.287	Ref	
	<37	42 (65.6)	1.4 (0.76,2.55)		1.1 (0.60,2.18)	0.681
Diabetes mellitus	No	123 (58.9)	Ref	NA		
	Yes	7 (100)	1 (NA)			

NA; Not applicable

For peer review only - http://bmjopen.bmj.com/site/about/guidelines.xhtml

DISCUSSION

In our study, the overall prevalence of retinopathy among women with hypertensive disorders of pregnancy was 60.1% with most of the lesions being Grade 1. Severe hypertension was independently associated with retinopathy.

The prevalence in our study is comparable to what was found in other studies done in Pakistan and Malaysia where the prevalence of retinopathy among women with hypertensive disorders of pregnancy was 51.9%-59% [17, 18]. Our result is lower than what was found in Kenya (90.8%)[19]. The difference may be because the study in Kenya considered postpartum women who had preeclampsia with severe features. Other studies have shown a lower prevalence of 12%-16.5% compared to what was found in our study [11, 20, 21]. The difference could be because in these studies a direct ophthalmoscope was the diagnostic tool used for retinopathy which could have possibly underestimated the participants with early retinal lesions on fundoscopy compared to our study where a fundus camera was used.

From our results, grade 1 retinopathy characterized by generalized arteriolar narrowing was the commonest retinal lesion while grade 3 and 4 retinopathy were the least observed among women with hypertensive disorders of pregnancy. This is similar to other studies where Grade 1 retinopathy was the commonest form of retinopathy and grade 3 and 4 retinopathy were the least common among women with hypertensive disorders of pregnancy [17, 18, 22]. However, a cross-sectional study done at Mulago National Hospital in Uganda among women with pre-eclampsia/eclampsia found that the commonest patterns of ocular manifestations were optic disc edema 19 (26.7%), retinal hemorrhages 17(22.9%) and cotton wool spots 16 (21.6%) [11]. This high proportion of severe retinal lesions compared to that observed in our study may be explained by the use of a direct ophthalmoscope with a possibility of underestimating early retinal lesions on fundoscopy while in our study a fundus camera was used with the ability to detect retinal lesions even at an early stage. Compared to our study, this study considered women who were presenting for maternity admission and these usually have severe disease while our study also considered women presenting in the outpatient antenatal clinics. Grade 1 retinal changes maybe due to constriction of retinal arterioles following elevated blood pressure [15]. Retinal changes seen in grade 3 and 4 could be due to endothelial damage, systemic inflammation, ischemia caused by hypoperfusion, or retinal edema caused by hyperfusion [23]. Presence of hypertensive retinopathy in pregnant women with hypertensive disorders of pregnancy could be an indicator of vascular changes in

For peer review only

placental circulation [11] and the severity of maternal fundus changes may indirectly indicate the status of the placental vasculature [15].

In our study, severe hypertension was associated with retinopathy among women with hypertensive disorders of pregnancy. This finding is similar to what was reported in others studies [14, 15, 18, 21, 24]. The retinal changes observed may be due to constriction of the retinal arterioles which occurs following the elevation of blood pressure [15]. The elevation in blood pressure involves a spectrum of retinal microvascular signs. These signs typically include; retinal arteriolar narrowing, arteriovenous nicking retinal hemorrhages, microaneurysms, and in severe cases, optic disc and macular edema [25].

Grandmultigravidity was associated with retinopathy although the association was not statistically significated. We have not found any previous studies that have demonstrated an association between grandmultigravidity and retinopathy.

Our study had some limitations. First, we only identified women who reported a known history of diabetes mellitus. However, we were not able to routinely screen the participants for diabetes or glucose intolerance during their pregnancy. Second, biochemical tests that determine hypertension with end organ damage were not done as we relied only on the blood pressure. Therefore we were not able to determine whether hypertension with end organ damage is a risk factor. Third, blood pressure was only done at one time point and therefore we could have missed out on participants who developed hypertension later in their pregnancy. Fourth, since we considered all pregnant women who presented with hypertension, we did not highlight the role of chronic hypertension in this study.

Conclusions

From our study, retinopathy among women with hypertensive disorders of pregnancy is common. Fundoscopy should form part of the routine examination for all women with hypertensive disorders of pregnancy to identify women with retinal lesions early enough and have them linked to care at the ophthalmology clinic for proper eye care management. Attention should also be given to women with severe hypertension and grandmultigravidas as these are more likely to have retinal lesions. There is a need for further research to document the long-term outcomes of women with retinopathy and hypertensive disorders of pregnancy as regards to their long-term vision.

For peer review only - http://bmjopen.bmj.com/site/about/guidelines.xhtml

Acknowledgment

We would like to convey our appreciation to the study participants for accepting to be part of this study, Ms. Naiga Patience and Ms. Florida Tusiimiraho our research assistants, Soar Research Foundation staff for support in data management, and to all the hospital administrators who offered permission to conduct this study in their hospitals.

Author Contributions

IZ, SR, CMM, TK, IBW, DA and HML conceived the study. IZ, CMM, TK, IBW and SR developed the data collection. DA, IZ and HML conducted the analysis. HML drafted the manuscript. All authors reviewed and approved the final manuscript.

Funding The authors have not declared a specific grant for this research from any funding agency in the public, commercial or not-for-profit sectors.

Research reported in this publication was supported by the Forgarty International Center of the National Institute of Health under award number D43TW011401 to HML. The content is solely the responsibility of the authors and does not necessarily represent the official views of the NIH.

The research reported here was supported by the Commonwealth Scholarship Commission and the Foreign, Commonwealth and Development Office in the UK to HML. I am grateful for their support. All views expressed here are those of the author(s) not the funding body. HML is funded by the Commonwealth Trust.

This research was supported, in part, by the NIHR Cambridge Biomedical Research Centre (BRC-1215-20014) to CMM. The views expressed are those of the authors and not necessarily those of the NIHR or the Department of Health and Social Care.
The funders had no role in study design, data collection, and analysis, decision to publish, or preparation of the manuscript.

Competing interests None declared

Patient and public involvement Patients and or the public were not involved in the design or conduct or reporting or dissemination plans of this research.

Patient consent for publication Not required

For peer review only - http://bmjopen.bmj.com/site/about/guidelines.xhtml

Ethical approval This study was approved by the Mbarara University of Science and Technology Research Ethics committee (IRB No: 16/09-19) and followed the tenets of the declaration of Helsinki. Written informed consent was obtained from all the participants that were enrolled into the study.

Data Availability Data are available upon request.

For peer review only

For peer review only - http://bmjopen.bmj.com/site/about/guidelines.xhtml

References

1. Errera, M.-H., R.P. Kohly, and L. Da Cruz *Pregnancy-associated retinal diseases and their management*. Survey of ophthalmology, 2013. **58**, 127-142.
2. Krishnakumar, S. and P. Chatterjee, *Fundus changes in preeclampsia.* Journal of Evolution of Medical and Dental Sciences, 2016. **5**(36): p. 2159-2163.
3. Bakhda, R.N., *Ocular manifestations of pregnancy induced hypertension.* The Official Scientific Journal of Delhi Ophthalmological Society, 2015. **26**(2): p. 88-92.
4. Khanom, R., et al., *Ocular Fundus Changes in Pregnancy Induced Hypertension.* Journal of Dhaka Medical College, 2020. **28**: p. 94-99.
5. Theodossiadis, P.G., et al., *Retinal disorders in preeclampsia studied with optical coherence tomography.* American journal of ophthalmology, 2002. **133**(5): p. 707-709.
6. Wolde, Z., H. Segni, and M. Woldie, *Hypertensive disorders of pregnancy in Jimma University specialized hospital.* Ethiopian journal of health sciences, 2011. **21**(3).
7. Azubuike, S. and I. Danjuma, *Hypertension in pregnancy among rural women in Katsina State, Nigeria.* Journal of Basic and Clinical Reproductive Sciences, 2017. **6**(1).
8. Kiondo, P., et al., *Adverse neonatal outcomes in women with pre-eclampsia in Mulago Hospital, Kampala, Uganda: a cross-sectional study.* The Pan African Medical Journal, 2014. **17**(Suppl 1).
9. Lugobe, H.M., et al., *490 Maternal mortality at a referral hospital in south western uganda: a 5 year descriptive analysis.* American Journal of Obstetrics & Gynecology, 2021. **224**(2): p. S311-S312.
10. Karki, P., et al., *Fundus Changes In Pregnancy Induced Hypertension.* Journal of Universal College of Medical Sciences, 2017. **5**(1): p. 42-44.
11. Bako, M.B., *The prevalence, patterns and factors associated with ocular manifestations of pre-eclampsia/eclampsia at Mulago National Referral Hospital.* 2018.
12. Gupta, A., et al., *Retinopathy in preeclampsia: association with birth weight and uric acid level.* Retina, 2008. **28**(8): p. 1104-1110.
13. Kaliaperumal, S., et al., *Fetal birthweight and diastolic blood pressure: association with retinopathy in severe preeclampsia.* European Journal of Ophthalmology, 2008. **18**(5): p. 809-812.
14. Tadin, I., et al., *Hypertensive retinopathy and pre-eclampsia.* Collegium antropologicum, 2001. **25**(1): p. 77-81.
15. Ye, L., et al., *Risk factors and pregnancy outcomes associated with retinopathy in patients presenting with severe preeclampsia: a retrospective cohort study.* Medicine, 2020. **99**(11).
16. Dutta, D. and H. Konar, *DC Dutta's Textbook of Gynecology*. 2014: JP Medical Ltd.
17. Janjua, M.I., S. Bano, and A. Raza, *Retinopathy in Pregnancy Induced Hypertension.* Pakistan Journal of Ophthalmology, 2015. **31**(4).
18. Reddy, S.C., *Fundus changes in pregnancy induced hypertension.* International Journal of Ophthalmology, 2012. **5**(6): p. 694.
19. Ayumba, K., Anne, *HIGH PREVALENCE AND SEVERITY OF POSTPARTUM RETINOVASCULAR CHANGES FOLLOWING PRE-ECLAMPSIA WITH SEVERE FEATURES COMPARED TO NORMAL PREGNANCY.* Journal of obstetrics & gynaecology of Eastern and Central Africa, 2019. **30**: p. 3-10.
20. karki, P., *Assiciation between preganncy induced hypertension fundus and fetal outcomes.* Nepal journal of ophthalmology, 2010. **2**.
21. Shah, A.P., et al., *Retinal changes in pregnancy-induced hypertension.* Medical Journal of Dr. DY Patil University, 2015. **8**(3): p. 304.
22. Rasdi, A.R., et al., *Hypertensive retinopathy and visual outcome in hypertensive disorders in pregnancy.* Med J Malaysia, 2011. **66**(1): p. 42-7.
23. Roos, N.M., et al., *Visual disturbances in (pre) eclampsia.* Obstetrical & gynecological survey, 2012. **67**(4): p. 242-250.
24. ZADE, M., et al., *RETINAL CHANGES IN PREGNANCY INDUCED-HYPERTENSION.* International Journal of Current Pharmaceutical Research, 2021: p. 18-20.

For peer review only

For peer review only - http://bmjopen.bmj.com/site/about/guidelines.xhtml

25. Bhargava, M., M. Ikram, and T.Y. Wong, *How does hypertension affect your eyes?* Journal of human hypertension, 2012. **26**(2): p. 71-83.

For peer review only

For peer review only - http://bmjopen.bmj.com/site/about/guidelines.xhtml

Fundus photos from 3inethra

1. Hypertensive Retinopathy grade 1: narrowing of the arterioles in both eyes.

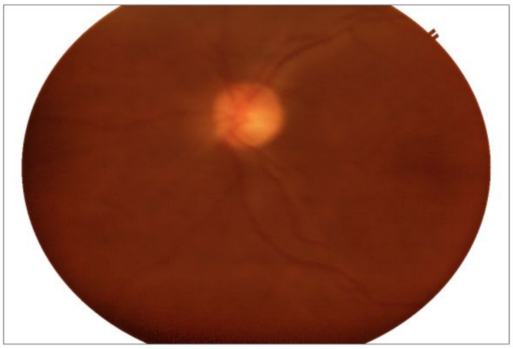

Posterior Left

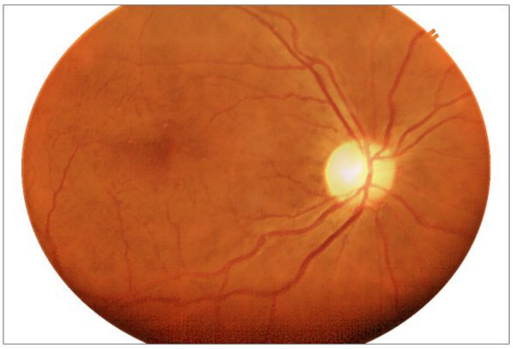

Posterior Right

2. Hypertensive retinopathy grade 4 Right eye :exsudates,dotsand blots hemorrhages and papilledema

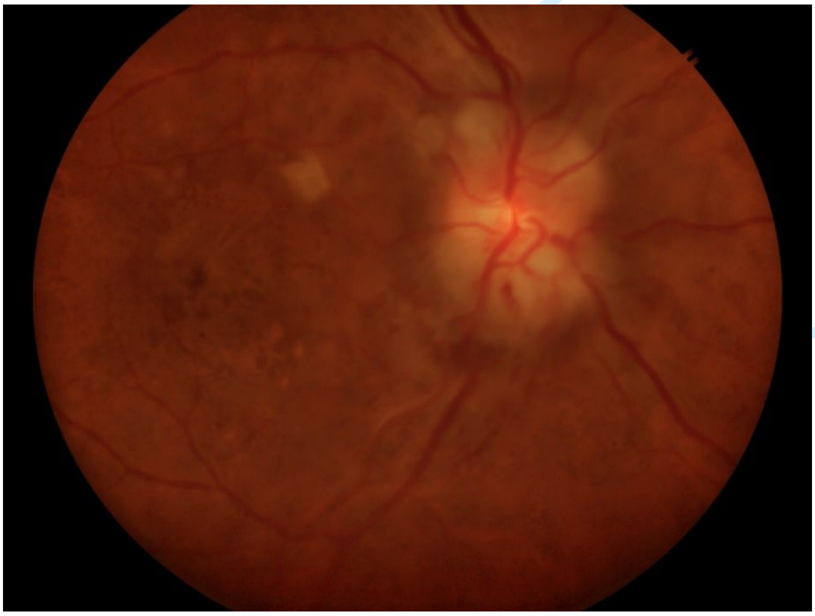

For peer review only - http://bmjopen.bmj.com/site/about/guidelines.xhtml

3. Hypertensive retinopathy grade 4 left eye: with exsudates ,flames shapes, dots and blots hemorrhages and papilledema.

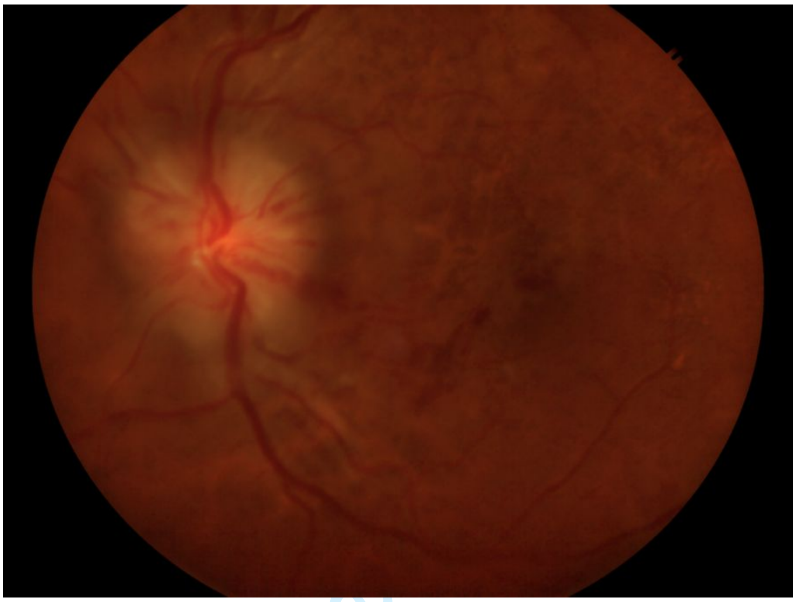

For peer review only

For peer review only - http://bmjopen.bmj.com/site/about/guidelines.xhtml

Reporting checklist for cross sectional study.

Based on the STROBE cross sectional guidelines.

Instructions to authors

Complete this checklist by entering the page numbers from your manuscript where readers will find each of the items listed below.

Your article may not currently address all the items on the checklist. Please modify your text to include the missing information. If you are certain that an item does not apply, please write "n/a" and provide a short explanation.

Upload your completed checklist as an extra file when you submit to a journal.

In your methods section, say that you used the STROBE cross sectionalreporting guidelines, and cite them as:

von Elm E, Altman DG, Egger M, Pocock SJ, Gotzsche PC, Vandenbroucke JP. The Strengthening the Reporting of Observational Studies in Epidemiology (STROBE) Statement: guidelines for reporting observational studies.

For peer review only

		Reporting Item	Page Number
**Title and abstract**			
Title	#1a	Indicate the study’s design with a commonly used term in the title or the abstract	1
Abstract	#1b	Provide in the abstract an informative and balanced summary of what was done and what was found	2
**Introduction**			
Background / rationale	#2	Explain the scientific background and rationale for the investigation being reported	4
Objectives	#3	State specific objectives, including any prespecified hypotheses	4
**Methods**			
Study design	#4	Present key elements of study design early in the paper	4
Setting	#5	Describe the setting, locations, and relevant dates, including periods of	5

		recruitment, exposure, follow-up, and data collection	
Eligibility criteria	#6a	Give the eligibility criteria, and the sources and methods of selection of participants.	5
	#7	Clearly define all outcomes, exposures, predictors, potential confounders, and effect modifiers. Give diagnostic criteria, if applicable	5 and 6
Data sources / measurement	#8	For each variable of interest give sources of data and details of methods of assessment (measurement). Describe comparability of assessment methods if there is more than one group. Give information separately for for exposed and unexposed groups if applicable.	5 and 6
Bias	#9	Describe any efforts to address potential sources of bias	7
Study size	#10	Explain how the study size was arrived at	6
Quantitative variables	#11	Explain how quantitative variables were handled in the analyses. If applicable, describe which groupings were chosen, and why	7
Statistical methods	#12a	Describe all statistical methods, including those used to control for confounding	7
Statistical methods	#12b	Describe any methods used to examine subgroups and interactions	n/a
Statistical methods	#12c	Explain how missing data were addressed	n/a
Statistical methods	#12d	If applicable, describe analytical methods taking account of sampling strategy	n/a
Statistical methods	#12e	Describe any sensitivity analyses	n/a
**Results**			
Participants	#13a	Report numbers of individuals at each stage of study—eg numbers potentially eligible, examined for eligibility, confirmed eligible, included in the study, completing follow-up, and analysed. Give information separately for for exposed and unexposed groups if applicable.	7
Participants	#13b	Give reasons for non-participation at each stage	n/a
Participants	#13c	Consider use of a flow diagram	n/a

For peer review only

Descriptive data	#14a	Give characteristics of study participants (eg demographic, clinical, social) and information on exposures and potential confounders. Give information separately for exposed and unexposed groups if applicable.	7
Descriptive data	#14b	Indicate number of participants with missing data for each variable of interest	n/a
Outcome data	#15	Report numbers of outcome events or summary measures. Give information separately for exposed and unexposed groups if applicable.	8 and 9
Main results	#16a	Give unadjusted estimates and, if applicable, confounder-adjusted estimates and their precision (eg, 95% confidence interval). Make clear which confounders were adjusted for and why they were included	9 and 10
Main results	#16b	Report category boundaries when continuous variables were categorized	n/a
Main results	#16c	If relevant, consider translating estimates of relative risk into absolute risk for a meaningful time period	n/a
Other analyses	#17	Report other analyses done—e.g., analyses of subgroups and interactions, and sensitivity analyses	n/a
**Discussion**			
Key results	#18	Summarise key results with reference to study objectives	11
Limitations	#19	Discuss limitations of the study, taking into account sources of potential bias or imprecision. Discuss both direction and magnitude of any potential bias.	12
Interpretation	#20	Give a cautious overall interpretation considering objectives, limitations, multiplicity of analyses, results from similar studies, and other relevant evidence.	11
Generalisability	#21	Discuss the generalisability (external validity) of the study results	11
**Other Information**			
Funding	#22	Give the source of funding and the role of the funders for the present study and, if applicable, for the original study on which the present article is based	13

The STROBE checklist is distributed under the terms of the Creative Commons Attribution License CC-BY. This checklist was completed on 05. June 2023 using https://www.goodreports.org/, a tool made by the EQUATOR Network in collaboration with Penelope.ai

For peer review only

BMJ Open

Retinopathy among women with hypertensive disorders of pregnancy attending hospitals in Mbarara city, south-western Uganda: A cross-sectional study

Journal:	*BMJ Open*
Manuscript ID	bmjopen-2023-076365.R1
Article Type:	Original research
Date Submitted by the Author:	13-Sep-2023
Complete List of Authors:	Zamaladi, Ibrahimu; Mbarara University of Science and Technology, Department of Ophthalmology<br>Ruvuma, Sam; Mbarara University of Science and Technology, Department of Ophthalmology<br>Mceniery, Carmel M. ; University of Cambridge<br>Kwaga, Teddy; Mbarara University of Science and Technology, Department of Ophthalmology<br>Wilkinson, Ian; Division of Experimental Medicine and Immunotherapeutics, Department of Medicine, University of Cambridge; Cambridge Clinical Trials Unit,<br>Atwine, Daniel; Soar Research Foundation, Department of Clinical Research; Mbarara University of Science and Technology, Department of Community Health<br>Lugobe, Henry Mark; University of Cambridge; Mbarara University of Science and Technology, Department of Obstetrics and Gynecology
**Primary Subject Heading**:	Ophthalmology
Secondary Subject Heading:	Cardiovascular medicine, Obstetrics and gynaecology
Keywords:	Hypertension < CARDIOLOGY, Maternal medicine < OBSTETRICS, Medical ophthalmology < OPHTHALMOLOGY

SCHOLARONE™
Manuscripts

I, the Submitting Author has the right to grant and does grant on behalf of all authors of the Work (as defined in the below author licence), an exclusive licence and/or a non-exclusive licence for contributions from authors who are: i) UK Crown employees; ii) where BMJ has agreed a CC-BY licence shall apply, and/or iii) in accordance with the terms applicable for US Federal Government officers or employees acting as part of their official duties; on a worldwide, perpetual, irrevocable, royalty-free basis to BMJ Publishing Group Ltd ("BMJ") its licensees and where the relevant Journal is co-owned by BMJ to the co-owners of the Journal, to publish the Work in this journal and any other BMJ products and to exploit all rights, as set out in our licence.

The Submitting Author accepts and understands that any supply made under these terms is made by BMJ to the Submitting Author unless you are acting as an employee on behalf of your employer or a postgraduate student of an affiliated institution which is paying any applicable article publishing charge ("APC") for Open Access articles. Where the Submitting Author wishes to make the Work available on an Open Access basis (and intends to pay the relevant APC), the terms of reuse of such Open Access shall be governed by a Creative Commons licence – details of these licences and which Creative Commons licence will apply to this Work are set out in our licence referred to above.

Other than as permitted in any relevant BMJ Author's Self Archiving Policies, I confirm this Work has not been accepted for publication elsewhere, is not being considered for publication elsewhere and does not duplicate material already published. I confirm all authors consent to publication of this Work and authorise the granting of this licence.

For peer review only

Retinopathy among women with hypertensive disorders of pregnancy attending hospitals in Mbarara city, south-western Uganda: A cross-sectional study

Ibrahimu Zamaladi[1], Sam Ruvuma [1], Carmel M. Mceniery[2] Teddy Kwaga[1], Ian B. Wilkinson[2], Daniel Atwine[3,4], Henry Mark Lugobe[2,5]*

[1]Department of Ophthalmology, Mbarara University of Science and Technology, Mbarara, Uganda.

[2]Division of Experimental Medicine and Immunotherapeutics, University of Cambridge, Cambridge, UK

[3]Department of Clinical Research, Soar Research Foundation, Mbarara, Uganda

[4]Department of Community Health, Mbarara University of Science and Technology, Mbarara, Uganda.

[5]Department of Obstetrics and Gynecology, Mbarara University of Science and Technology, Mbarara, Uganda.

*Corresponding author:

Henry Mark Lugobe

PO Box 1410, Mbarara

henrylugobe@must.ac.ug

Word count: 2444

Keywords: Retinopathy, Hypertensive disorders of pregnancy, Pregnancy, Uganda

For peer review only

Abstract

Objective: Retinopathy is one of the complications occurring among women with hypertensive disorders of pregnancy. We sought to determine the prevalence and factors associated with retinopathy among women with hypertensive disorders of pregnancy in southwestern Uganda.

Design: This was a hospital based cross-sectional study from November 2019 to March 2020.

Setting: Three selected hospitals in Mbarara city, south-western Uganda,

Participants: The study included all pregnant women with hypertensive disorders of pregnancy.

Primary and secondary outcome measures: The participants were screened for retinopathy using a fundus camera. Data on participant's socio-demographics, obstetrics, and medical factors were collected. The prevalence of retinopathy was determined and multivariable logistic regression was used to determine the independent factors associated with retinopathy.

Results: A total of 216 women with hypertensive disorders of pregnancy were enrolled in this study. The prevalence of retinopathy was 60.1% (130/216). The commonest retinal lesions were Grade 1 retinopathy (narrowing of arterioles) accounting for 86.9% (113/130), Grade 3 (retinal hemorrhages) was present in 10% (13/130) of women, and Grade 4 (papilledema) in 3% (4/130). In an adjusted analysis, severe hypertension was significantly associated with retinopathy (aOR=2.8; 95%CI: 1.36, 5.68). Grandmultigravida women were also associated with retinopathy (aOR=2.4; 95%CI: 0.99, 5.72) with a tendency towards significancy, p=0.051.

Conclusions: In our study, retinopathy was common among women with hypertensive disorders of pregnancy. Women presenting with severe hypertension were likely to have retinopathy. There is a need to integrate screening for retinopathy in the care cascade of women with hypertensive disorders of pregnancy.

For peer review only

Strengths and limitations of this study

- A portable non-mydriatic fundus camera was used to examine for retinopathy.
- Blood pressure was only done at one time point.
- Routine screening of participants for diabetes or glucose intolerance during their pregnancy was not done.
- Biochemical tests to determine presence of hypertension with end organ damage were not done.

For peer review only

For peer review only - http://bmjopen.bmj.com/site/about/guidelines.xhtml

Introduction

Retinopathy in pregnancy is a term that defines the retinal pathologies seen uniquely in pregnancy or more commonly in conditions that may worsen or alter during pregnancy as a result of hematologic and metabolic change [1]. During pregnancy, elevated blood pressure poses various risks including placental abruption, intrauterine growth retardation, premature delivery, future cardiovascular diseases, and injury to other organs including the eye [2]. Ocular involvement is common in a majority of cases of hypertensive disorders of pregnancy. Common symptoms are blurry vision, photopsia, scotomas, and diplopia [3]. Visual disturbances and retinopathy in hypertensive disorders of pregnancy seem to be a frequent phenomenon [4]. The Global prevalence of hypertensive disorders of pregnancy ranges between 5-10% and fundus changes are seen in 40-100% of these patients [5]. In Africa, hospital-based prevalence of hypertensive disorders of pregnancy is 8% in Ethiopia [6] and 17% in Nigeria [7]. In Uganda, hypertensive disorders of pregnancy accounted for 12% of maternal deaths in 4 referral hospitals [8] while at Mbarara regional referral hospital they accounted for 22% of the maternal deaths [9].

Studies in Malaysia and India showed that 40% and 59% of women with hypertensive disorders of pregnancy respectively presented with retinal changes while in Nepal it was 14% [10]. In Uganda, a study done at Mulago National Hospital showed a prevalence of 16.5% of retinal changes in preeclampsia/eclampsia patients [11]. Retinopathy among women with hypertensive disorders of pregnancy is associated with fetal birth weight, serum uric acid, proteinuria, severe hypertension and maternal age[12-15].

However, there is a paucity of information on retinopathy among women with hypertensive disorders of pregnancy in sub-saharan Africa where hypertension in pregnancy is associated with significant maternal morbidity and mortality. We sought to determine the prevalence and factors associated with retinopathy among women with hypertensive disorders of pregnancy in south-western Uganda.

Materials and Methods

Study design and setting

We conducted a hospital-based, cross-sectional study of women with hypertensive disorders of pregnancy, at three selected hospitals in south-western Uganda from November 2019 to March

For peer review only - http://bmjopen.bmj.com/site/about/guidelines.xhtml

2020. These hospitals were Mbarara Regional Referral hospital, Mayanja Memorial hospital, and Divine Mercy hospital. Mbarara regional referral hospital is a government-owned referral hospital that offers free services as well as a teaching hospital for the medical school of Mbarara University of Science and Technology. The hospital conducts about 10,000 deliveries every year. Mayanja Memorial Hospital is a private hospital with a capacity of 60 beds while Divine Mercy Hospital is also a private hospital with a capacity of 22 beds. All these facilities offer antenatal, labor, delivery, and post-natal care services. These facilities receive referrals from sixteen districts in south-western Uganda.

We received ethical approval from the Mbarara University of Science and Technology Research Ethics Committee (NO 16/09-19). All the study participants provided written informed consent.

Study participants

Our study population included all pregnant women at ≥ 20 weeks of gestation with hypertensive disorders of pregnancy who were receiving care either as in-patients at the antenatal ward or as out-patients at the antenatal clinic. The blood pressure was measured at admission or during the antenatal clinic visit. Hypertension was defined as systolic blood pressure ≥140mmHg or diastolic blood pressure ≥ 90mmHg. Women with ocular diseases that prevented adequate fundus examination, such as cataracts, corneal opacities, and ocular trauma, were excluded from the study. All mothers presenting at the maternity ward or antenatal clinic had their blood pressure checked as part of routine clinical care and eligible participants were consecutively enrolled in the study.

Variables and data sources

Hypertensive disorders of pregnancy were classified into eclampsia, pre-eclampsia, gestational hypertension, chronic hypertension, and chronic hypertension with superimposed preeclampsia, according to consensus definitions [16]. All pregnant women who had hypertensive disorders of pregnancy either diagnosed as inpatient or outpatient were screened for retinopathy. The primary outcome for our study was retinopathy and was determined using Keith and Wagner classification [17].

Grade 1: Mild generalized arterial attenuation, particularly of small branches, with broadening of the arteriolar light reflex.

For peer review only - http://bmjopen.bmj.com/site/about/guidelines.xhtml

Grade 2: Marked generalized narrowing and focal attenuation of arterioles associated with arterio-venous nipping/narrowing (Salus' sign).

Grade 3: Grade 2 changes plus copper-wiring of arterioles, banking of veins distal to arteriovenous crossings (Bonnet sign), tapering of veins on either side of the crossings (Gunn sign). Flame-shaped hemorrhages, cotton wool spots, and hard exudates are also present.

Grade 4: All changes of Grade 3 plus silver wiring of arterioles and papilledema.

Uncorrected visual acuity was measured using a Snellen chart and E-chart. Anterior segment examination was done using a torch while fundoscopy was done using both a direct ophthalmoscope (brand Keeler) after dilating the pupil with tropicamide 1%, and used a portable non-mydriatic fundus camera (Forus 3nethra non-mydriatic, Forus Health Pvt Ltd, Bengaluru, India). Fundus changes were recorded and all fundus photos were interpreted by a consultant retinal specialist of Mbarara University Referral Hospital Eye Center. The fundus pictures were graded for quality before KW grading.

The independent variables included socio-demographic characteristics (age, level of education, address, district of origin, and occupation), medical factors (known history of diabetes mellitus, hypertension), and obstetric factors (gravidity, severity of hypertension (systolic blood pressure ≥160mmHg and or diastolic blood pressure ≥100mmHg), and gestational age). At enrolment into the study, participants were interviewed and information was obtained using an interviewer-administered questionnaire.

Sample size calculation

We calculated a total sample size of 216 participants using the formula for cross-sectional sample size calculations of Kish Leslie with the following assumptions.

$n= (Z\alpha^2 p\ (1-p))/\delta^2$

Where

n= sample size estimate.

p= prevalence (16.5 %) as estimated by a study done at Mulago hospital [11]

δ= acceptable margin of error 5% (0.05).

$Z\alpha$= standard normal deviation at 95% Confidence Interval 1.96.

1-p= the probability of having retinopathy (1-0.17) = 0.83

$n= (1.96^2 \times 0.17(1-0.17)) / 0.05^2$

n= 216 participants

For peer review only - http://bmjopen.bmj.com/site/about/guidelines.xhtml

Statistical analysis

Data were entered into Microsoft excel 2010 and were imported into STATA 15.0 software (college station Texas USA) for analysis. Descriptive statistics were presented as means and standard deviations for continuous variables and proportions for categorical variables. The proportion of patients with retinopathy was calculated as the number of participants with retinal changes over the total number of study participants and expressed as a percentage with its corresponding 95% Confidence Interval (CI). Retinal changes were classified according to KEITH WAGNER classification into grade 1, grade 2, grade 3, and grade 4, and the proportion of each grade was presented in a frequency table. Bivariate analysis using pearson Chi-square test and logistic regression analysis was performed comparing participant factors with retinopathy. At bivariate analysis, variables with statistically significant association with retinopathy ($p<0.05$), those with $p<0.1$ and those with biological plausibility with retinopathy e.g age were included in the multivariable logistic regression model. A manual stepwise backward elimination was used to build the final model. The independent factors associated with retinopathy in the final multivariable model were reported together with their adjusted odds ratios and 95% confidence interval. The level of significance was $p<0.05$.

Patient and public involvement Patients and or the public were not involved in the design or conduct or reporting or dissemination plans of this research.

Results

Study Profile

A total of 5008 pregnant women attended Mbarara Regional Referral Hospital, Mayanja memorial Hospital, and Divine Mercy Hospital during the study period. Of these, 216 women with hypertensive disorders of pregnancy were consented and enrolled in the study. A total of 208 (96.3%), 6 (2.8%) and 2 (0.9%) were recruited from Mbarara Regional Referral Hospital, Mayanja Hospital and Divine Mercy Hospital respectively. Of the participants, 88.4% (191/216) had preeclampsia, 10.2% (22/216) had eclampsia and 1.4% (3/216) had gestational hypertension.

Participants' Characteristics

For peer review only - http://bmjopen.bmj.com/site/about/guidelines.xhtml

Table 1 shows the sociodemographic and medical characteristics of women enrolled in the study. Participants had a mean age of 27.6±6.37 years with majority aged less than 35 years (81.7%), Ugandan (90.3%) and either unemployed or peasants (74.5%). Participants predominantly had 37 or more gestational weeks (70.4%) and were either multigravida or grand multigravida mothers (72%). One in four had severe hypertension (26.4%) at the time of the study. Majority of participants (214/216) had good uncorrected visual acuity of 6/6-6/18, and only 1/216 had severe visual impairment. A third of the participants presented with blurry vision. There was no case of complete blindness in our study.

Table 1: Baseline characteristics of participants

**Characteristics**		**n (%)**
Age in years, mean(SD)		27.6 (6.37)
Age category	<25	77 (35.4)
	25-34	100 (46.3)
	35-46	39 (18.1)
Nationality	Ugandan	195 (90.3)
	Non-Ugandan	21 (9.7)
Occupation	Unemployed	84 (38.9)
	Peasant	77 (35.6)
	Business	30 (13.9)
	Professional	17 (7.9)
	Laborer	8 (3.7)
Known history of diabetes mellitus	No	209 (96.8)
	Yes	7 (3.2)
Severe hypertension	No	159 (73.6)
	Yes	57 (26.4)
Gravidity	Primigravidas	61 (28.2)
	Multigravida	111 (51.4)
	Grand multigravida	44 (20.4)
Gestational age (weeks)	<37	64 (29.6)
	≥37	152 (70.4)
Ocular symptoms	No complaints	42(19.4)
	Blurry vision	71 (32.9)
	Reduced vision	7 (3.3)
	Photopsia	2 (0.9)
	Inability to focus	1 (0.5)
	Visual field defect	1 (0.5)
Visual acuity	6/6-6/18	214 (99.0)
	<6/18-6/60	1 (0.5)
	6/60-3/60	1 (0.5)

For peer review only - http://bmjopen.bmj.com/site/about/guidelines.xhtml

Prevalence of hypertensive retinopathy among pregnant women

Of the 216 women with hypertensive disorders of pregnancy attending hospitals in Mbarara city and enrolled in the study, 130 had retinopathy, giving a prevalence of 60.2% (95% CI: 53.5-66.5). No age-specific disparities where noted with regard to prevalence of retinopathy, p=0.136 as shown in Table 2.

Table 2. Prevalence of hypertensive retinopathy among pregnant women

**Prevalence type**	**N**	**n**	**% (95% CI)**	**p value**
Overall	216	130	60.2 (53.5-66.5)	NA
Age-specific				0.136
<25	77	44	57.1 (45.7-67.9)	
25-34	100	57	57.0 (47.0-66.5)	
35-46	39	29	74.4 (57.8-86.0)	

NA; Not applicable

Of the 130 women with retinopathy, 113 (86.9%) presented with arteriolar narrowing (Figure 1). Severe retinopathy characterized by retinal hemorrhages and papilledema (Figure 2) was also recorded. No one had Grade 2 retinopathy and no retinal detachment as shown in Table 3.

Table 3: Description of retinal lesions found among women with hypertensive disorders of pregnancy according to Keith and Wagner classification

**Retinal lesions (n=130)**		**n (%)**
Grade 1	Arteriolar narrowing	113 (86.92)
Grade 3	Dot and blot hemorrhages	6 (4.62)
	Flamme shape hemorrhages	3 (2.30)
	Cotton wool spots	2 (1.54)
	Exudates	2 (1.54)
Grade 4	Papilledema	2 (1.54)
	Maculoedema	2 (1.54)

For peer review only - http://bmjopen.bmj.com/site/about/guidelines.xhtml

Factors associated with retinopathy among women with hypertensive disorders of pregnancy

Table 4 shows results of bivariate and multivariate analysis. In bivariate analysis, the only factors that were significantly associated with retinopathy were gravidity (p=0.049) and severe hypertension (p=0.005).

In multivariate analysis, severe hypertension was the only factor significantly associated with retinopathy after adjusting for age, gravidity and gestational age, p=0.005. The odds of having retinopathy where 2.8 times higher among women with severe hypertension as compared to those without, OR=2.8; 95%CI: 1.36, 5.68. Also, the odds of having retinopathy were 2.4 times higher among grandmultigravida women as compared to those with multigravida women, OR=2.4; 95%CI: 0.99, 5.72, although this did not attain statistical significance, p=0.051.

Table 4: Factors associated with retinopathy among women with hypertensive disorders of pregnancy.

Variable	Categories	Retinopathy n (%)	Crude OR (95% CI)	p-value	Adjusted OR (95% CI)	p-value
Age	<25	44 (57.1)	Ref	0.124	Ref	
	25-34	57 (57.0)	0.9 (0.55,1.81)		0.9 (0.42,1.72)	0.655
	35-46	29 (74.4)	2.2 (0.93,5.08)		1.5 (0.54,4.18)	0.435
Nationality	Ugandan	116 (59.5)	Ref	0.519		
	Non-ugandan	14 (66.7)	1.4 (0.53,3.53)			
Occupation	Unemployed	56 (66.7)	1.8 (0.62,5.11)	0.4723		
	Peasant	41 (53.3)	1.0 (0.35,2.90)			
	Business	19 (63.3)	1.5 (0.46,5.14)			
	Professional	9 (52.9)	Ref			
	Laborer	5 (62.5)	1.5 (0.27,8.27)			
Severe hypertension	No	87 (54.7)	Ref	0.005	Ref	
	Yes	43 (75.4)	2.5 (1.29, 5.01)		2.8 (1.36,5.68)	0.005
Gravidity	Multigravid	60 (54.1)	Ref	0.049	Ref	

For peer review only - http://bmjopen.bmj.com/site/about/guidelines.xhtml

	Primigravida	37 (60.7)	1.3 (0.69,2.47)		1.2 (0.61,2.54)	0.556
	Grandmultigravida	33 (75.0)	2.5 (1.17,5.55)		2.4 (0.99,5.72)	0.051
Gestational age	≥37	88 (57.9)	Ref	0.287	Ref	
	<37	42 (65.6)	1.4 (0.76,2.55)		1.1 (0.60,2.18)	0.681
Diabetes mellitus	No	123 (58.9)	Ref	NA		
	Yes	7 (100)	1 (NA)			

NA; Not applicable

For peer review only

For peer review only - http://bmjopen.bmj.com/site/about/guidelines.xhtml

DISCUSSION

In our study, the overall prevalence of retinopathy among women with hypertensive disorders of pregnancy was 60.1% with most of the lesions being Grade 1. Severe hypertension was independently associated with retinopathy.

The prevalence in our study is comparable to what was found in other studies done in Pakistan and Malaysia where the prevalence of retinopathy among women with hypertensive disorders of pregnancy was 51.9%-59% [17, 18]. Our result is lower than what was found in Kenya (90.8%)[19]. The difference may be because the study in Kenya considered postpartum women who had preeclampsia with severe features. Other studies have shown a lower prevalence of 12%-16.5% compared to what was found in our study [11, 20, 21]. The difference could be because in these studies a direct ophthalmoscope was the diagnostic tool used for retinopathy which could have possibly underestimated the participants with early retinal lesions on fundoscopy compared to our study where a fundus camera was used.

From our results, grade 1 retinopathy characterized by generalized arteriolar narrowing was the commonest retinal lesion while grade 3 and 4 retinopathy were the least observed among women with hypertensive disorders of pregnancy. This is similar to other studies where Grade 1 retinopathy was the commonest form of retinopathy and grade 3 and 4 retinopathy were the least common among women with hypertensive disorders of pregnancy [17, 18, 22]. However, a cross-sectional study done at Mulago National Hospital in Uganda among women with pre-eclampsia/eclampsia found that the commonest patterns of ocular manifestations were optic disc edema 19 (26.7%), retinal hemorrhages 17(22.9%) and cotton wool spots 16 (21.6%) [11] . This high proportion of severe retinal lesions compared to that observed in our study may be explained by the use of a direct ophthalmoscope with a possibility of underestimating early retinal lesions on fundoscopy while in our study a fundus camera was used with the ability to detect retinal lesions even at an early stage. Compared to our study, this study considered women who were presenting for maternity admission and these usually have severe disease while our study also considered women presenting in the outpatient antenatal clinics. Grade 1 retinal changes maybe due to constriction of retinal arterioles following elevated blood pressure [15]. Retinal changes seen in grade 3 and 4 could be due to endothelial damage, systemic inflammation, ischemia caused by hypoperfusion, or retinal edema caused by hyperfusion [23]. Presence of hypertensive retinopathy in pregnant women with hypertensive disorders of pregnancy could be an indicator of vascular changes in

For peer review only - http://bmjopen.bmj.com/site/about/guidelines.xhtml

placental circulation [11] and the severity of maternal fundus changes may indirectly indicate the status of the placental vasculature [15].

In our study, severe hypertension was associated with retinopathy among women with hypertensive disorders of pregnancy. This finding is similar to what was reported in others studies [14, 15, 18, 21, 24]. The retinal changes observed may be due to constriction of the retinal arterioles which occurs following the elevation of blood pressure [15]. The elevation in blood pressure involves a spectrum of retinal microvascular signs. These signs typically include; retinal arteriolar narrowing, arteriovenous nicking retinal hemorrhages, microaneurysms, and in severe cases, optic disc and macular edema [25].

Grandmultigravidity was associated with retinopathy although the association was not statistically significant. We have not found any previous studies that have demonstrated an association between grandmultigravidity and retinopathy.

Our study had some limitations. First, we only identified women who reported a known history of diabetes mellitus. However, we were not able to routinely screen the participants for diabetes or glucose intolerance during their pregnancy. Second, biochemical tests that determine hypertension with end organ damage were not done as we relied only on the blood pressure. Therefore we were not able to determine whether hypertension with end organ damage is a risk factor. Third, blood pressure was only done at one time point and therefore we could have missed out on participants who developed hypertension later in their pregnancy. Fourth, since we considered all pregnant women who presented with hypertension, we did not highlight the role of chronic hypertension in this study.

Conclusions

From our study, retinopathy among women with hypertensive disorders of pregnancy is common. Fundoscopy should form part of the routine examination for all women with hypertensive disorders of pregnancy to identify women with retinal lesions early enough and have them linked to care at the ophthalmology clinic for proper eye care management. Attention should also be given to women with severe hypertension and grandmultigravidas as these are more likely to have retinal lesions. There is a need for further research to document the long-term outcomes of women with retinopathy and hypertensive disorders of pregnancy as regards to their long-term vision.

For peer review only - http://bmjopen.bmj.com/site/about/guidelines.xhtml

Acknowledgment

We would like to convey our appreciation to the study participants for accepting to be part of this study, Ms. Naiga Patience and Ms. Florida Tusiimiraho our research assistants, Soar Research Foundation staff for support in data management, and to all the hospital administrators who offered permission to conduct this study in their hospitals.

Author Contributions

IZ, SR, CMM, TK, IBW, DA and HML conceived the study. IZ, CMM, TK, IBW and SR developed the data collection. DA, IZ and HML conducted the analysis. HML drafted the manuscript. All authors reviewed and approved the final manuscript.

Funding The authors have not declared a specific grant for this research from any funding agency in the public, commercial or not-for-profit sectors.

Research reported in this publication was supported by the Forgarty International Center of the National Institute of Health under award number D43TW011401 to HML. The content is solely the responsibility of the authors and does not necessarily represent the official views of the NIH.

The research reported here was supported by the Commonwealth Scholarship Commission and the Foreign, Commonwealth and Development Office in the UK to HML. I am grateful for their support. All views expressed here are those of the author(s) not the funding body. HML is funded by the Commonwealth Trust.

This research was supported, in part, by the NIHR Cambridge Biomedical Research Centre (BRC-1215-20014) to CMM. The views expressed are those of the authors and not necessarily those of the NIHR or the Department of Health and Social Care.
The funders had no role in study design, data collection, and analysis, decision to publish, or preparation of the manuscript.

Competing interests None declared

Patient and public involvement Patients and or the public were not involved in the design or conduct or reporting or dissemination plans of this research.

Patient consent for publication Not required

For peer review only - http://bmjopen.bmj.com/site/about/guidelines.xhtml

Ethical approval This study was approved by the Mbarara University of Science and Technology Research Ethics committee (IRB No: 16/09-19) and followed the tenets of the declaration of Helsinki. Written informed consent was obtained from all the participants that were enrolled into the study.

Data Availability Data are available upon request.

For peer review only

Figure 1 showing hypertensive retinopathy grade 1: narrowing of the arterioles in both eyes.

Figure 2 showing hypertensive retinopathy grade 4 in both eyes: exudates, flame shaped hemorrhages and papilledema.

For peer review only - http://bmjopen.bmj.com/site/about/guidelines.xhtml

References

1. Errera, M.-H., R.P. Kohly, and L. Da Cruz *Pregnancy-associated retinal diseases and their management*. Survey of ophthalmology, 2013. **58**, 127-142.
2. Krishnakumar, S. and P. Chatterjee, *Fundus changes in preeclampsia.* Journal of Evolution of Medical and Dental Sciences, 2016. **5**(36): p. 2159-2163.
3. Bakhda, R.N., *Ocular manifestations of pregnancy induced hypertension.* The Official Scientific Journal of Delhi Ophthalmological Society, 2015. **26**(2): p. 88-92.
4. Khanom, R., et al., *Ocular Fundus Changes in Pregnancy Induced Hypertension.* Journal of Dhaka Medical College, 2020. **28**: p. 94-99.
5. Theodossiadis, P.G., et al., *Retinal disorders in preeclampsia studied with optical coherence tomography.* American journal of ophthalmology, 2002. **133**(5): p. 707-709.
6. Wolde, Z., H. Segni, and M. Woldie, *Hypertensive disorders of pregnancy in Jimma University specialized hospital.* Ethiopian journal of health sciences, 2011. **21**(3).
7. Azubuike, S. and I. Danjuma, *Hypertension in pregnancy among rural women in Katsina State, Nigeria.* Journal of Basic and Clinical Reproductive Sciences, 2017. **6**(1).
8. Kiondo, P., et al., *Adverse neonatal outcomes in women with pre-eclampsia in Mulago Hospital, Kampala, Uganda: a cross-sectional study.* The Pan African Medical Journal, 2014. **17**(Suppl 1).
9. Lugobe, H.M., et al., *490 Maternal mortality at a referral hospital in south western uganda: a 5 year descriptive analysis.* American Journal of Obstetrics & Gynecology, 2021. **224**(2): p. S311-S312.
10. Karki, P., et al., *Fundus Changes In Pregnancy Induced Hypertension.* Journal of Universal College of Medical Sciences, 2017. **5**(1): p. 42-44.
11. Beatrice, B., *Magnitude, Pattern and Factors Associated with Ocular Manifestations in Pre-eclampsia/Eclampsia at Mulago National Referral Hospital.* Uganda. J Ophthalmol Adv Res, 2021. **2**(1): p. 1-7.
12. Gupta, A., et al., *Retinopathy in preeclampsia: association with birth weight and uric acid level.* Retina, 2008. **28**(8): p. 1104-1110.
13. Kaliaperumal, S., et al., *Fetal birthweight and diastolic blood pressure: association with retinopathy in severe preeclampsia.* European Journal of Ophthalmology, 2008. **18**(5): p. 809-812.
14. Tadin, I., et al., *Hypertensive retinopathy and pre-eclampsia.* Collegium antropologicum, 2001. **25**(1): p. 77-81.
15. Ye, L., et al., *Risk factors and pregnancy outcomes associated with retinopathy in patients presenting with severe preeclampsia: a retrospective cohort study.* Medicine, 2020. **99**(11).
16. Dutta, D. and H. Konar, *DC Dutta's Textbook of Gynecology*. 2014: JP Medical Ltd.
17. Janjua, M.I., S. Bano, and A. Raza, *Retinopathy in Pregnancy Induced Hypertension.* Pakistan Journal of Ophthalmology, 2015. **31**(4).
18. Reddy, S.C., *Fundus changes in pregnancy induced hypertension.* International Journal of Ophthalmology, 2012. **5**(6): p. 694.
19. Ayumba, K., Anne, *HIGH PREVALENCE AND SEVERITY OF POSTPARTUM RETINOVASCULAR CHANGES FOLLOWING PRE-ECLAMPSIA WITH SEVERE FEATURES COMPARED TO NORMAL PREGNANCY.* Journal of obstetrics & gynaecology of Eastern and Central Africa, 2019. **30**: p. 3-10.
20. karki, P., *Assiciation between preganncy induced hypertension fundus and fetal outcomes.* Nepal journal of ophthalmology, 2010. **2**.
21. Shah, A.P., et al., *Retinal changes in pregnancy-induced hypertension.* Medical Journal of Dr. DY Patil University, 2015. **8**(3): p. 304.
22. Rasdi, A.R., et al., *Hypertensive retinopathy and visual outcome in hypertensive disorders in pregnancy.* Med J Malaysia, 2011. **66**(1): p. 42-7.

For peer review only - http://bmjopen.bmj.com/site/about/guidelines.xhtml

23. Roos, N.M., et al., *Visual disturbances in (pre) eclampsia.* Obstetrical & gynecological survey, 2012. **67**(4): p. 242-250.
24. ZADE, M., et al., *RETINAL CHANGES IN PREGNANCY INDUCED-HYPERTENSION.* International Journal of Current Pharmaceutical Research, 2021: p. 18-20.
25. Bhargava, M., M. Ikram, and T.Y. Wong, *How does hypertension affect your eyes?* Journal of human hypertension, 2012. **26**(2): p. 71-83.

For peer review only

For peer review only - http://bmjopen.bmj.com/site/about/guidelines.xhtml

Figure 1: Hypertensive retinopathy grade 1: narrowing of the arterioles in both eyes.

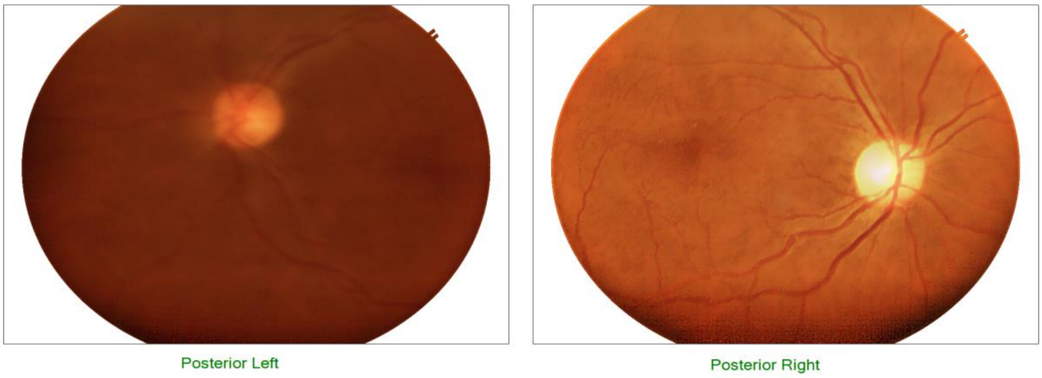

For peer review only

Figure 2: Hypertensive retinopathy grade 4 in both eyes: exudates, flame shaped hemorrhages and papilledema.

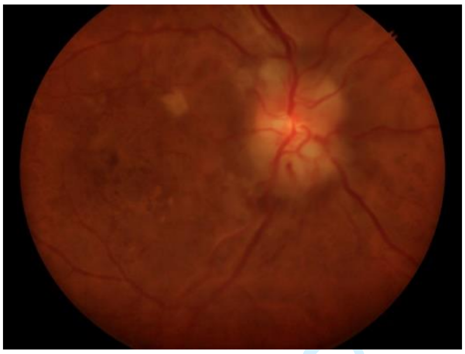

Right eye

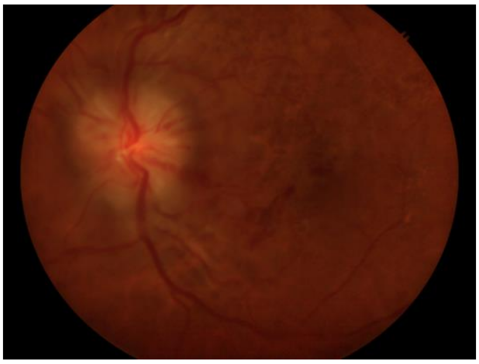

Left eye

For peer review only - http://bmjopen.bmj.com/site/about/guidelines.xhtml

Reporting checklist for cross sectional study.

Based on the STROBE cross sectional guidelines.

Instructions to authors

Complete this checklist by entering the page numbers from your manuscript where readers will find each of the items listed below.

Your article may not currently address all the items on the checklist. Please modify your text to include the missing information. If you are certain that an item does not apply, please write "n/a" and provide a short explanation.

Upload your completed checklist as an extra file when you submit to a journal.

In your methods section, say that you used the STROBE cross sectionalreporting guidelines, and cite them as:

von Elm E, Altman DG, Egger M, Pocock SJ, Gotzsche PC, Vandenbroucke JP. The Strengthening the Reporting of Observational Studies in Epidemiology (STROBE) Statement: guidelines for reporting observational studies.

For peer review only

		Reporting Item	Page Number
**Title and abstract**			
Title	#1a	Indicate the study's design with a commonly used term in the title or the abstract	1
Abstract	#1b	Provide in the abstract an informative and balanced summary of what was done and what was found	2
**Introduction**			
Background / rationale	#2	Explain the scientific background and rationale for the investigation being reported	4
Objectives	#3	State specific objectives, including any prespecified hypotheses	4
**Methods**			
Study design	#4	Present key elements of study design early in the paper	4
Setting	#5	Describe the setting, locations, and relevant dates, including periods of	5

		recruitment, exposure, follow-up, and data collection	
Eligibility criteria	#6a	Give the eligibility criteria, and the sources and methods of selection of participants.	5
	#7	Clearly define all outcomes, exposures, predictors, potential confounders, and effect modifiers. Give diagnostic criteria, if applicable	5 and 6
Data sources / measurement	#8	For each variable of interest give sources of data and details of methods of assessment (measurement). Describe comparability of assessment methods if there is more than one group. Give information separately for for exposed and unexposed groups if applicable.	5 and 6
Bias	#9	Describe any efforts to address potential sources of bias	7
Study size	#10	Explain how the study size was arrived at	6
Quantitative variables	#11	Explain how quantitative variables were handled in the analyses. If applicable, describe which groupings were chosen, and why	7
Statistical methods	#12a	Describe all statistical methods, including those used to control for confounding	7
Statistical methods	#12b	Describe any methods used to examine subgroups and interactions	n/a
Statistical methods	#12c	Explain how missing data were addressed	n/a
Statistical methods	#12d	If applicable, describe analytical methods taking account of sampling strategy	n/a
Statistical methods	#12e	Describe any sensitivity analyses	n/a
**Results**			
Participants	#13a	Report numbers of individuals at each stage of study—eg numbers potentially eligible, examined for eligibility, confirmed eligible, included in the study, completing follow-up, and analysed. Give information separately for for exposed and unexposed groups if applicable.	7
Participants	#13b	Give reasons for non-participation at each stage	n/a
Participants	#13c	Consider use of a flow diagram	n/a

For peer review only

Descriptive data	#14a	Give characteristics of study participants (eg demographic, clinical, social) and information on exposures and potential confounders. Give information separately for exposed and unexposed groups if applicable.	7
Descriptive data	#14b	Indicate number of participants with missing data for each variable of interest	n/a
Outcome data	#15	Report numbers of outcome events or summary measures. Give information separately for exposed and unexposed groups if applicable.	8 and 9
Main results	#16a	Give unadjusted estimates and, if applicable, confounder-adjusted estimates and their precision (eg, 95% confidence interval). Make clear which confounders were adjusted for and why they were included	9 and 10
Main results	#16b	Report category boundaries when continuous variables were categorized	n/a
Main results	#16c	If relevant, consider translating estimates of relative risk into absolute risk for a meaningful time period	n/a
Other analyses	#17	Report other analyses done—e.g., analyses of subgroups and interactions, and sensitivity analyses	n/a
**Discussion**			
Key results	#18	Summarise key results with reference to study objectives	11
Limitations	#19	Discuss limitations of the study, taking into account sources of potential bias or imprecision. Discuss both direction and magnitude of any potential bias.	12
Interpretation	#20	Give a cautious overall interpretation considering objectives, limitations, multiplicity of analyses, results from similar studies, and other relevant evidence.	11
Generalisability	#21	Discuss the generalisability (external validity) of the study results	11
**Other Information**			
Funding	#22	Give the source of funding and the role of the funders for the present study and, if applicable, for the original study on which the present article is based	13

The STROBE checklist is distributed under the terms of the Creative Commons Attribution License CC-BY.
This checklist was completed on 05. June 2023 using https://www.goodreports.org/, a tool made by the EQUATOR Network in collaboration with Penelope.ai

For peer review only
